# More Than Just Bone Pain: A Case of Paget’s Disease, Seropositive Rheumatoid Arthritis, and Metabolic Dysfunction-Associated Steatohepatitis (MASH) in a Patient With Elevated Alkaline Phosphatase (ALP)

**DOI:** 10.7759/cureus.88191

**Published:** 2025-07-17

**Authors:** Paige Webeler, Grace E Kim, Rahul Paryani

**Affiliations:** 1 Internal Medicine, Baylor Scott & White Medical Center, Fort Worth, USA; 2 Internal Medicine, Medical Associates of North Texas, Fort Worth, USA

**Keywords:** alkaline phosphatase (alp), chronic pain, metabolic dysfunction-associated steatohepatitis (mash), paget’s disease of bone (pdb), rheumatoid arthritis

## Abstract

Paget’s disease of bone is a chronic skeletal disorder characterized by disorganized bone remodeling. Although often asymptomatic, it is frequently first detected through elevated alkaline phosphatase (ALP). Given ALP’s nonspecific nature, its elevation requires a comprehensive evaluation for hepatic, biliary, and bone pathology. We present a diagnostic challenge involving persistent ALP elevation in a patient ultimately found to have coexisting Paget’s disease, metabolic dysfunction-associated steatohepatitis (MASH), and seropositive rheumatoid arthritis (RA).

## Introduction

Paget’s disease of bone is a chronic disorder characterized by disorganized bone remodeling, which can lead to bone pain, deformities, and fractures. While often asymptomatic, it may first be detected through incidental laboratory findings such as elevated alkaline phosphatase (ALP). The differential diagnosis of elevated ALP includes hepatic, biliary, and bone pathology, necessitating a comprehensive clinical evaluation [1]. We present a case of a patient with systemic symptoms and persistent ALP elevation who was ultimately diagnosed with Paget’s disease of bone, metabolic dysfunction-associated steatohepatitis (MASH), and seropositive rheumatoid arthritis (RA). To our knowledge, there are few reported cases in the literature describing the co-occurrence of these three conditions.

## Case presentation

A 62-year-old male with a history of hypertension, type 2 diabetes mellitus, hyperlipidemia, obstructive sleep apnea, stage 3 chronic kidney disease secondary to diabetic and hypertensive nephropathy, and chronic back pain was evaluated for persistently elevated ALP for six months and chronic musculoskeletal pain. His chronic back pain followed a motor vehicle accident requiring C4-C5 fusion and a subsequent workplace injury that necessitated L5-S1 fusion. Despite these interventions, he reported diffuse pain involving the neck, hips, knees, ankles, and fingers, along with morning stiffness, tingling of the fingertips, fatigue, dry eyes, and unilateral hearing loss.

Laboratory workup showed persistently elevated ALP with normal aspartate aminotransferase (AST) and alanine transaminase (ALT). Physical examination was notable for swelling and tenderness of the bilateral shoulders, knees, ankles, cervical spine, and metacarpophalangeal joints.

The elevated ALP was evaluated with comprehensive laboratory and imaging studies. Results showed elevated ALP, parathyroid hormone (PTH), antinuclear antibody (ANA), and gamma-glutamyl transferase (GGT) (Table 1). Hepatitis serologies, anti-cyclic citrullinated peptide (anti-CCP), anti-mitochondrial antibody (AMA), and tissue transglutaminase (TTG) were negative.

**Table 1:** Selected laboratory values

Lab name	Lab value	Normal lab value	Units
Alkaline phosphatase (ALP)	247	45-117	U/mL
Aspartate aminotransferase (AST)	40	8-33	U/L
Alanine aminotransferase (ALT)	42	7-55	U/L
Bilirubin	0.6	0.1-1.2	mg/dL
Ferritin	152	12-300	ng/mL
Parathyroid hormone (PTH)	166	10-65	pg/mL
Calcium	9.0	8.5-10.2	mg/dL
Creatinine	2.1	0.7-1.3	mg/dL
Anti-nuclear antibody (ANA)	1:160	<1:80	-
Gamma-glutamyl transferase (GGT)	125	5-40	U/mL

Abdominal ultrasound showed no biliary dilation or acute abnormalities. Liver biopsy revealed grade 1 inflammation with stage 0 fibrosis, consistent with MASH. However, due to persistent elevation of ALP and GGT over the following years, the patient underwent further hepatology evaluation. A positive anti-smooth muscle antibody (ASMA, 1:80) was noted, while the remainder of the chronic liver disease panel was unremarkable. Liver synthetic function remained preserved. FibroScan revealed F2 (moderate) fibrosis. Given the diagnosis of MASH, the patient was started on tirzepatide and dapagliflozin, with subsequent weight loss of 29 kilograms. 

**Figure 1:**
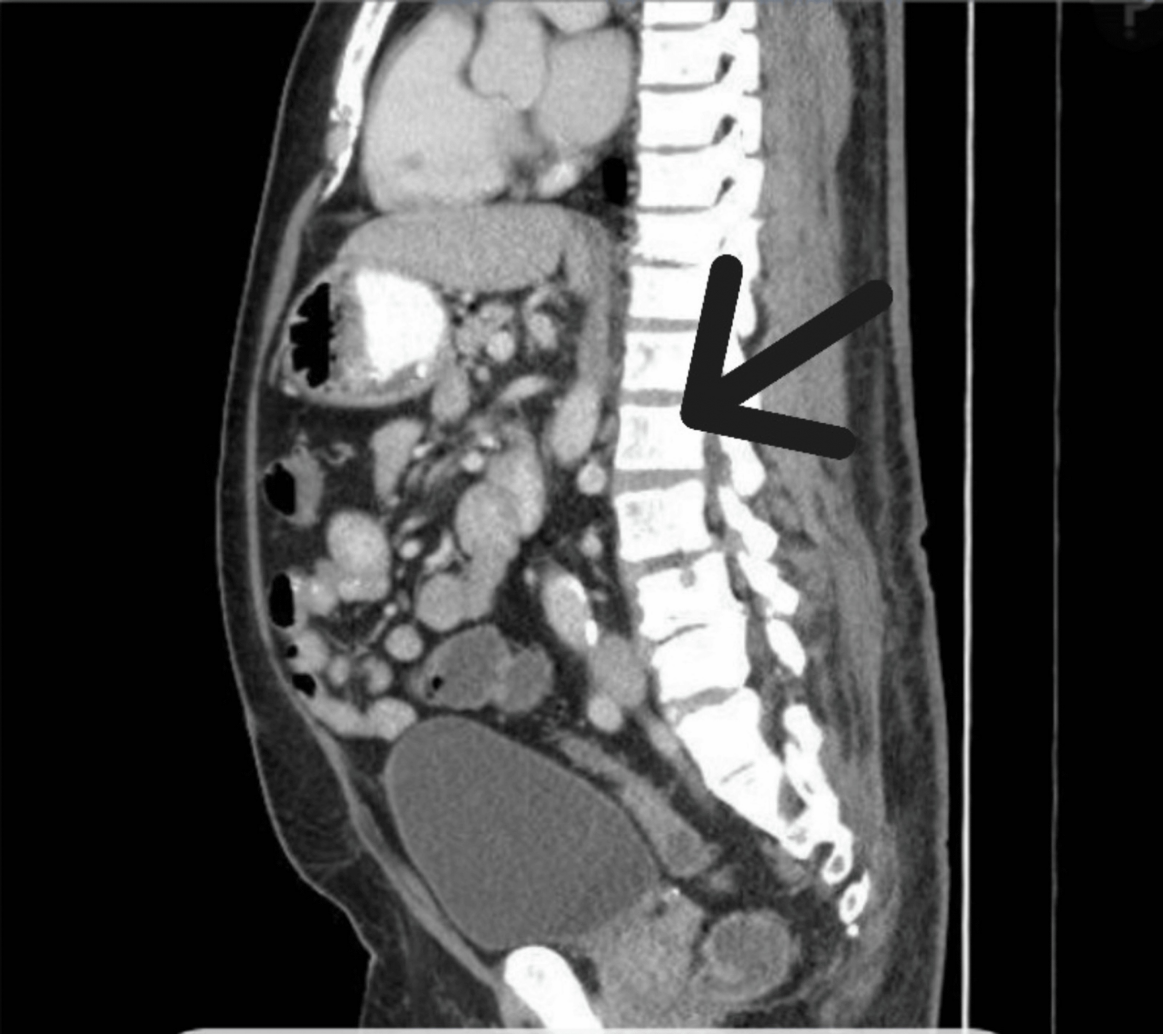
Contrast-enhanced CT of the abdomen and pelvis showing cortical sclerosis and bony expansion involving the right hemipelvis, sacrum, and lower lumbar vertebrae, consistent with Paget’s disease. Arrow indicates area of degeneration and sclerosis of affected vertebral bodies.

**Figure 2:**
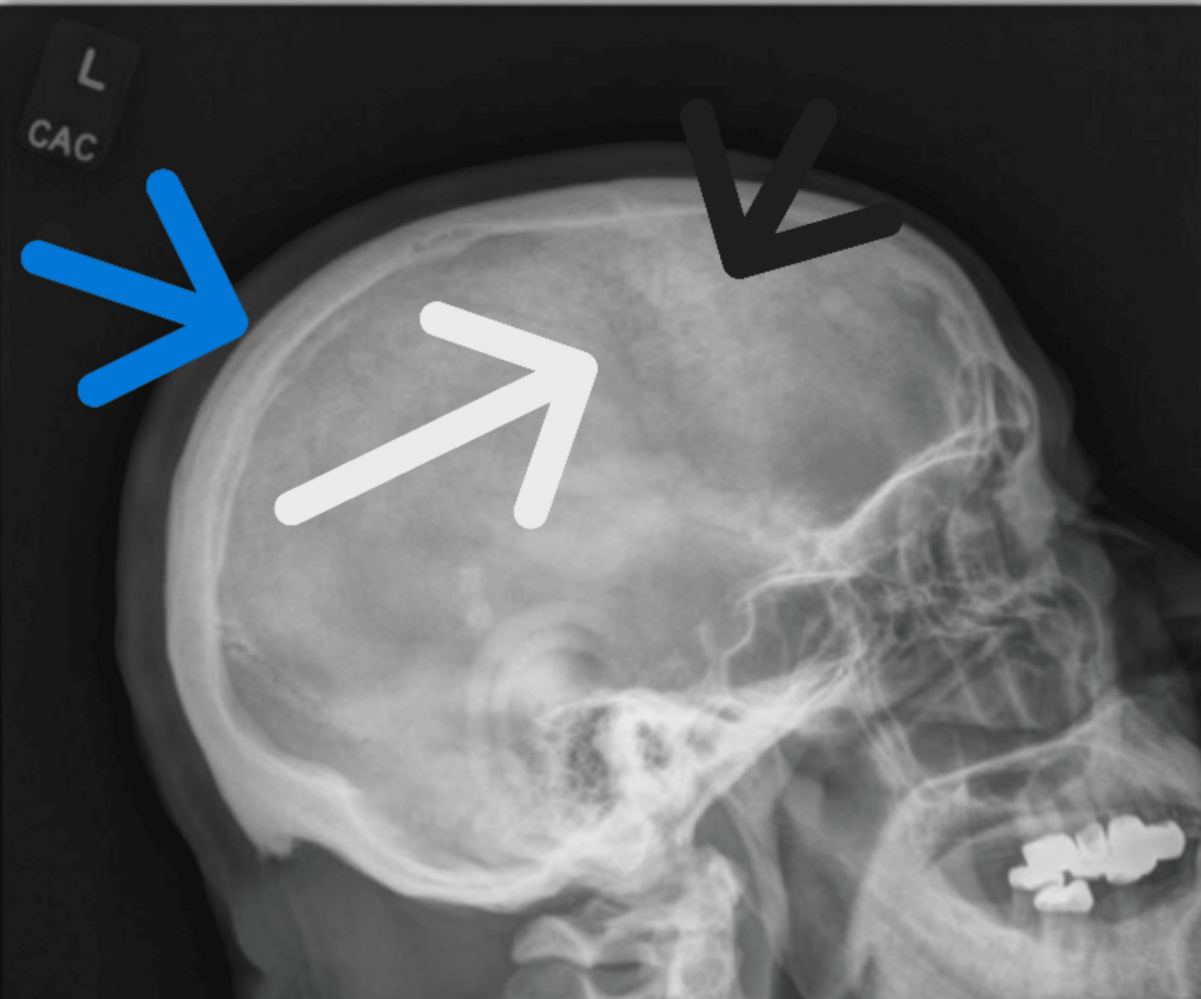
Skull radiograph demonstrating diffuse patchy mixed sclerotic and lytic lesions. Findings include a thickened calvarium (blue arrow), an area of sclerosis (black arrow), and a lytic lesion (white arrow).

A CT of the abdomen and pelvis, obtained to evaluate thrombocytopenia and suspected splenomegaly, demonstrated diffuse cortical and trabecular sclerosis with bony expansion of the right hemipelvis, sacrum, and lower lumbar vertebrae-findings consistent with Paget’s disease (Figure 1). A skull radiograph was obtained to further evaluate bony involvement, revealing diffuse, patchy, mixed sclerotic and lytic lesions (Figure 2). ALP isoenzyme fractionation confirmed a bone-predominant source (68.9%). Audiology testing revealed right-sided sensorineural hearing loss. 

Given his multifocal joint pain, prolonged morning stiffness, and thrombocytopenia, further autoimmune workup was pursued. Rheumatoid factor (RF) and ANA were elevated, and the clinical picture was consistent with seropositive rheumatoid arthritis. He was started on hydroxychloroquine 200 mg daily, later increased to twice daily, with significant improvement in symptoms. Given his underlying renal dysfunction, the patient was initiated on a reduced dose of bisphosphonate therapy for Paget’s disease.

## Discussion

Paget’s disease is a chronic skeletal disorder characterized by excessive and disorganized bone remodeling, most commonly involving the pelvis, spine, and femur. It primarily affects individuals over age 50, with a prevalence of 1.5% [1]. Genetic mutations, including SQSTM1, have been associated with the disease [1]. The pathophysiology involves hyperactive osteoclasts stimulating disorganized osteoblast activity, resulting in structurally weak but sclerotic bone [2].

While often asymptomatic, approximately 30% of patients experience bone pain, fractures, or hearing loss. Diagnosis is based on characteristic radiologic findings and elevated bone turnover markers such as ALP or type I collagen N-terminal telopeptide [2-3]. First-line treatment includes bisphosphonates or calcitonin to suppress osteoclast activity [1].

ALP is a non-specific enzyme produced primarily by the liver and bone, and its elevation can reflect either cholestasis or increased osteoblastic activity. Evaluating the specific cause of ALP elevation is essential due to its broad differential diagnosis. GGT, a more specific marker for hepatobiliary disease, can help differentiate the source of ALP elevation [4]. PTH is elevated in up to 20% of patients with Paget’s due to increased bone turnover demands [5].

In this case, elevated ALP in the context of normal transaminases, bone majority ALP isoenzyme fractionation, and characteristic skeletal imaging led to a diagnosis of Paget’s disease. Type I collagen N-terminal telopeptide testing was not available. The presence of hepatic steatosis (MASH) complicated the initial workup, highlighting the need for comprehensive imaging and isoenzyme analysis. The elevated GGT was attributed to MASH. Although ASMA was mildly positive, liver biopsy findings were not consistent with autoimmune hepatitis, effectively ruling it out as a cause of the elevated GGT and ALP. The patient’s multifocal joint pain, morning stiffness, and serologic findings supported a concurrent diagnosis of seropositive RA.

For the management of MASH, the patient was started on tirzepatide and dapagliflozin following shared decision-making. Emerging evidence supports the use of SGLT2 inhibitors in improving hepatic steatosis, reducing liver fibrosis, and enhancing metabolic parameters in patients with NAFLD and type 2 diabetes [6]. In addition, the SYNERGY-NASH trial demonstrated that tirzepatide significantly increased the rate of steatohepatitis resolution without worsening fibrosis in patients with stage 2 or 3 fibrosis, highlighting its potential as a therapeutic option in MASH management [7]. 

## Conclusions

Paget’s disease should be considered in patients with unexplained ALP elevation and characteristic skeletal changes. This case highlights the complexity of diagnosing ALP elevation in the setting of multiple overlapping conditions, including MASH and rheumatoid arthritis. The patient was treated with bisphosphonates for Paget’s disease, tirzepatide for MASH, and hydroxychloroquine for seropositive rheumatoid arthritis. It also highlights the importance of interdisciplinary collaboration and continuing workup when clinical symptoms extend beyond a single diagnosis. Given the limited literature describing the coexistence of Paget’s disease and RA, this case aims to raise clinicians' awareness of this rare but meaningful diagnostic overlap.
